# Pericytes recruited by CCL28 promote vascular normalization after anti-angiogenesis therapy through RA/RXRA/ANGPT1 pathway in lung adenocarcinoma

**DOI:** 10.1186/s13046-024-03135-3

**Published:** 2024-07-29

**Authors:** Ying Chen, Zhiyong Zhang, Fan Pan, Pengfei Li, Weiping Yao, Yuxi Chen, Lei Xiong, Tingting Wang, Yan Li, Guichun Huang

**Affiliations:** 1 The State Key Laboratory of Pharmaceutical Biotechnology, Chemistry and Biomedicine Innovation Center (ChemBIC), Division of Immunology, Medical School, Nanjing University, Nanjing, 210093 China; 2 Jiangsu Key Laboratory of Molecular Medicine, Division of Immunology, Medical School, Nanjing University, Nanjing, 210093 China; 3 Medical Schoolof, Nanjing University, Nanjing, Jiangsu 210093 China; 4 Department of Respiratory Critical Care Medicine, Affiliated Hospital of Medical School, Nanjing Drum Tower Hospital, Nanjing University, Nanjing, Jiangsu 210008 China; 5 Department of Cardio-Thoracic Surgery, Affiliated Hospital of Medical School, Jinling Hospital, Nanjing University, Nanjing, Jiangsu 210008 China; 6 Department of Medical Oncology, Affiliated Hospital of Medical School, Jinling Hospital, Nanjing University, Nanjing, Jiangsu 210008 China; 7 Department of Oncology, Medical School, Zhongda Hospital, Southeast University, Nanjing, 210009 China

**Keywords:** Anti-angiogenesis therapy, Vascular normalization, Pericytes, CCL28, Lung adenocarcinoma, Retinoic acid

## Abstract

**Background:**

It has been proposed that anti-angiogenesis therapy could induce tumor "vascular normalization" and further enhance the efficacy of chemotherapy, radiotherapy, target therapy, and immunotherapy for nearly twenty years. However, the detailed molecular mechanism of this phenomenon is still obscure.

**Method:**

Overexpression and knockout of CCL28 in human lung adenocarcinoma cell line A549 and murine lung adenocarcinoma cell line LLC, respectively, were utilized to establish mouse models. Single-cell sequencing was performed to analyze the proportion of different cell clusters and metabolic changes in the tumor microenvironment (TME). Immunofluorescence and multiplex immunohistochemistry were conducted in murine tumor tissues and clinical biopsy samples to assess the percentage of pericytes coverage. Primary pericytes were isolated from lung adenocarcinoma tumor tissues using magnetic-activated cell sorting (MACS). These pericytes were then treated with recombinant human CCL28 protein, followed by transwell migration assays and RNA sequencing analysis. Changes in the secretome and metabolome were examined, and verification of retinoic acid metabolism alterations in pericytes was conducted using quantitative real-time PCR, western blotting, and LC–MS technology. Chromatin immunoprecipitation followed by quantitative PCR (ChIP-qPCR) was employed to validate the transcriptional regulatory ability and affinity of RXRα to specific sites at the ANGPT1 promoter.

**Results:**

Our study showed that after undergoing anti-angiogenesis treatment, the tumor exhibited a state of ischemia and hypoxia, leading to an upregulation in the expression of CCL28 in hypoxic lung adenocarcinoma cells by the hypoxia-sensitive transcription factor CEBPB. Increased CCL28 could promote tumor vascular normalization through recruiting and metabolic reprogramming pericytes in the tumor microenvironment. Mechanistically, CCL28 modified the retinoic acid (RA) metabolism and increased ANGPT1 expression via RXRα in pericytes, thereby enhancing the stability of endothelial cells.

**Conclusion:**

We reported the details of the molecular mechanisms of "vascular normalization" after anti-angiogenesis therapy for the first time. Our work might provide a prospective molecular marker for guiding the clinical arrangement of combination therapy between anti-angiogenesis treatment and other therapies.

**Supplementary Information:**

The online version contains supplementary material available at 10.1186/s13046-024-03135-3.

## Introduction

Lung cancer stands as the foremost cause of mortality among patients with malignant tumors, with lung adenocarcinoma being a predominant pathological subtype [1, 2]. Angiogenesis is a crucial hallmark of lung adenocarcinoma, and significant strides have been made in applying anti-angiogenesis therapy for its treatment [3]. Extensive research data indicates that anti-angiogenic tumor therapy exhibits the potential to augment the effectiveness of diverse therapeutic modalities, including chemotherapy, radiotherapy, tyrosine kinase inhibitors (TKIs), and immunotherapy. Nevertheless, a subset of patients fails to derive benefits from the combined approach of anti-angiogenic therapy and other anti-tumor treatments. Unraveling the synergistic mechanisms underlying anti-angiogenic therapy holds substantial clinical implications for optimizing combination therapies.

Since the 2000s, there has been a fundamental shift in the understanding of anti-angiogenic therapy for tumors, transitioning from the initial concept of solely inhibiting angiogenesis to inducing vascular normalization in tumors [4]. Presently, there is a consensus that optimizing the effectiveness of additional anti-tumor treatments through anti-angiogenic therapy is imperative. This notion is central to anti-angiogenic drugs' capability to induce transient vascular normalization in tumor blood vessels [5]. Preliminary investigations undertaken by our research group propose that anti-angiogenic drugs, employing diverse mechanisms of action, can prompt vascular normalization in lung adenocarcinoma [6–9]. The balance between pro-angiogenesis and anti-angiogenesis factors was postulated as the key molecular base of vascular normalization [10]. However, uncertainties persist regarding the timing and the limited duration of vascular normalization post-treatment. Addressing this issue is critical, as the temporal aspects of vascular normalization and its short-lived nature need clarification [7–9, 11, 12]. The pressing clinical challenge involves establishing protocols to monitor and regulate vascular normalization in lung adenocarcinoma, offering valuable guidance for developing combined anti-angiogenic therapies with other anti-tumor treatment strategies. A comprehensive understanding of the molecular mechanisms underpinning anti-angiogenic therapy-induced vascular normalization in lung adenocarcinoma forms the foundational basis for resolving this intricate clinical dilemma.

Pericytes, one of the structural cells found in capillary vessels, envelop the basal membrane of endothelial cells and interact with vascular endothelial cells to ensure the stability and functionality of microvessels [13, 14]. Commonly used markers for identifying pericytes include platelet-derived growth factor receptor β (PDGFRβ), chondroitin sulfate proteoglycan 4 (CSPG4, also known as NG2), and alpha-smooth muscle actin (α-SMA). Several reports suggest that platelet-derived growth factor B (PDGFB) can promote vascular normalization in colon cancer and malignant melanoma by recruiting pericytes [15]. However, PDGFB does not perform this function in pericytes in lung adenocarcinoma [16]. The mechanism of pericyte recruitment in lung adenocarcinoma following anti-angiogenesis therapy remains unclear.

Human CC motif chemokine ligands 28 (CCL28) can recruit various cell types, including lymphatic endothelial cells [13], T cells, and plasma cells, through its receptors CCR3 or CCR10. Apart from its chemotactic properties, CCL28 has been implicated in promoting tumor development in ovarian cancer [17], gastric cancer [18], liver cancer, and lung adenocarcinoma [19]. Under hypoxic conditions, tumor cells mobilize various cell types using different chemokines to induce angiogenesis in response to nutritional and oxygen requirements. Notably, hypoxia induces the expression of CCL28 in ovarian cancer, which, in turn, enhances angiogenesis by recruiting regulatory T cells (Tregs) [17]. In contrast, CCL28 has been identified as a negative regulator of tumor growth and bone invasion in oral squamous cell carcinoma [20]. These diverse findings suggest that CCL28 may play distinct roles in various tumors. While one study discovered that CCL28 increased the proliferation, migration, and secretion of IL-6 and HGF in oral fibroblasts [21], the functional role of CCL28 in pericytes in lung adenocarcinoma has not been elucidated. Furthermore, previous studies have reported that CCL28 was involved in angiogenesis in various diseases, including tumors, skin wound healing [22], and rheumatoid arthritis [23]. However, little is known about the mechanism of CCL28 in tumor vascular normalization.

Our previous report highlighted that CCL28 can moderately enhance the angiogenesis in lung adenocarcinoma [19]. Interestingly, we found a normalized vasculature in CCL28 highly expressed tumors. The current study delves into the previously undisclosed connection between pericytes and CCL28 in lung adenocarcinoma. Both in vivo and in vitro experiments collectively illustrate that CCL28 derived from tumor cells is pivotal in advancing vascular normalization by mobilizing and reprogramming pericytes.

## Materials and methods

### Cancer cell lines

The human lung adenocarcinoma cell line (A549, SPC-A1, and H1975) and mouse Lewis lung cancer cell line (LLC) were purchased from the Shanghai Institute of Biochemistry and Cell Biology (SIBCB) and maintained in our lab. Lung cancer cells were cultured in RPMI-1640, or DMEM medium (Gibco, LifeTech, USA) supplemented with 10% fetal bovine serum (FBS), 1% antibiotics (100U/ml penicillin and 100 μg/ml streptomycin) at 37℃ in a humidified 5% CO_2_ atmosphere.

### Isolation and identification of pericytes

Pericytes were isolated and cultured as we previously described [24]. Fresh lung cancer samples were collected from patients in Jinling Hospital (Nanjing, China). Tissue samples were cut into small blocks of approximately 1 ~ 2 mm in diameter, digested with trypsin and 0.5% collagenase, and filtered through the cell strainer to obtain single-cell suspension. PDGFRβ + cells were isolated by PE-PDGFRβ antibody and anti-PE magnetic beads (Miltenyi Biotec,130–123-772). The separated cells were cultured in F12K medium (Gibco, LifeTech, USA) containing 10% fetal bovine serum (Gibco, Life Tech, USA) with 100 U/mL penicillin and 100 μg/mL streptomycin. After two or three passages, pericytes were identified by morphology and immunofluorescence staining for α-Smooth Muscle Actin (α-SMA, CST, #19,245), platelet-derived growth factor receptor β (PDGFRβ, Abcam, ab69506) and chondroitin sulfate proteoglycan 4 (NG2, Abcam, ab129051). Patients with incomplete data were excluded to evaluate the clinical effect. Written informed consent was obtained from all subjects before collecting the samples. All the methods followed the institutional guidelines and were approved by the Ethical Review Committee of Jinling Hospital, Nanjing, China(2022DZGZR-QH-005).

### Hypoxic culture model and RNA sequence assay

**Fig. 1:**
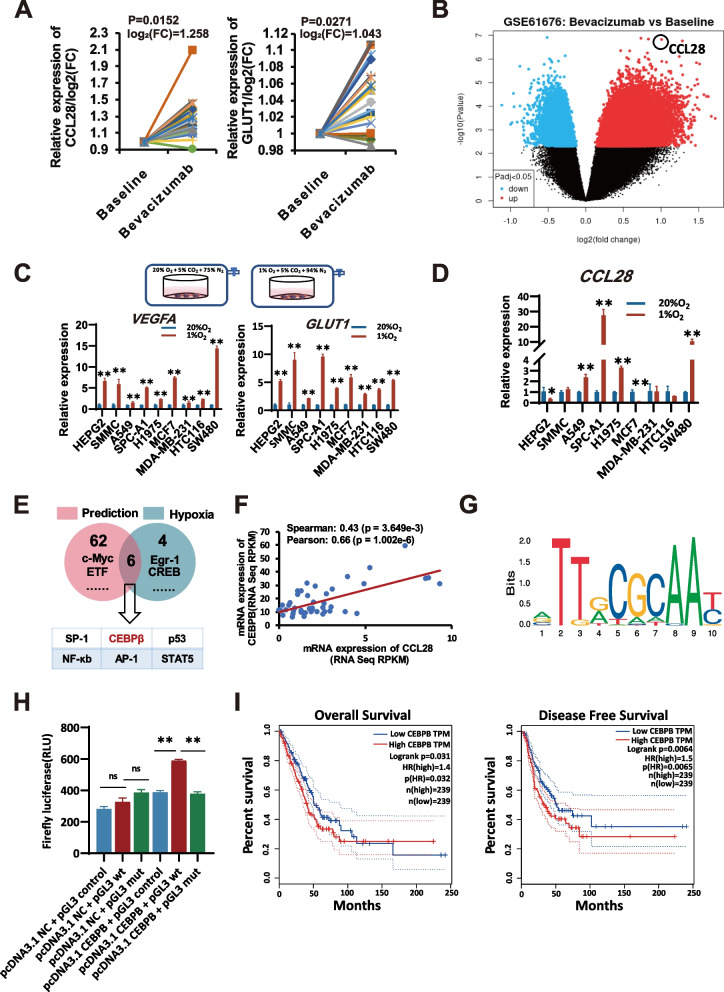
CCL28 expression is upregulated after anti-angiogenesis therapy by hypoxia-sensitive transcription factor CEBPB in lung adenocarcinoma

As previously reported, the hypoxic cell culture model was established with hypoxic chambers in our lab [19]. Briefly, lung adenocarcinoma cells were cultured under two different oxygen concentrations, 1% and 20%, respectively. The model was testified by the expression changes of HIF-1α and its regulated genes, such as GLUT1 and VEGFA (Fig. 1C). Pericytes were cultured under different stimulation. After culturing for 24 h, the cells were collected, and the total amount of RNA or protein was extracted for western blot or quantitative PCR. RNA sequence assay was applied to detect the gene expression differences of pericytes cultured with or without the stimulation of CCL28 (MCE, HY-P7250) under hypoxia.

### RNA extraction and quantitative PCR

Total RNA was extracted from different cell lines using Trizol reagent (Invitrogen, USA). Subsequently, reverse transcription and quantitative PCR were performed using SYBR Green with an ABI StepOne Plus System (Applied Biosystems, Life Tech, USA). Relative gene expression was calculated by the ΔΔCt method based on glyceraldehyde-3-phosphate dehydrogenase (GAPDH) or β-actin levels. All reactions were run in triplicate.

### Luciferase reporter assay

Wild-type (pGL3 wt, 5’-TGATTATGCAATGG-3') and mutant (pGL3 mut, 5’-ACTAATACGTTACC-3') promoters of the CCL28 gene were constructed into pGL3 firefly luciferase reporter plasmid vector purchased from Nanjing Realgene Bio-Technology Company (Nanjing China). The pRL-TK vector expressing renilla luciferase was used as an internal control. The reporter plasmid was co-transfected with pRL-TK vector and CEBPB expressing pcDNA3.1 vector or control vector into A549 cell. Dual-Luciferase Reporter Assay System (Promega, USA) was used to detect the luciferase activity after 48-h incubation.

### Chromatin Immunoprecipitation (ChIP)

Briefly, pericytes treated with or without recombinant human CCL28 or CCR3 (R&D Systems, MAB155-100) neutralizing antibodies were collected and fixed by adding a cross-linking agent, formaldehyde, to stabilize the interactions between chromatin proteins and DNA. Subsequently, chromatin immunoprecipitation was conducted to enrich DNA fragments bound by chromatin proteins. After cross-link reversal and DNA purification, real-time quantitative PCR (qPCR) technology is employed to measure enriched DNA, determining the relative abundance at specific gene loci.

### Liquid Chromatography-Mass Spectrometry (LC–MS)

Liquid chromatography-mass spectrometry was applied to measure retinoic acid (RA, HY-14649) in pericytes. Briefly, after treatment with or without CCL28, pericytes were subjected to enzymatic digestion, washed three times with PBS, flash-frozen in liquid nitrogen for one minute, and then stored at -80 °C. When performing the detection, begin by thawing the sample at 4 °C. Next, add 0.5 mL of methanol solution, followed by 10 min of sonication and 30 min of shaking for extraction. Subsequently, place the centrifuge tube in a low-temperature centrifuge and centrifuge for 10 min at 4 °C and 12,000 rpm. Collect 800μL of the supernatant, evaporate it, and then add 100μL of methanol solution. Finally, retain 80 μL of the supernatant for subsequent liquid chromatography-mass spectrometry analysis. A standard curve is generated to calculate the sample's content using a retinoic acid standard (yuanye Bio-Technology).

### Single-cell RNA sequencing

Lewis lung cancer cells overexpressing CCL28 were inoculated in C57BL/6 mice subcutaneously (1 × 10^6^ cells per mouse). When the tumor grew to about 100 mm^3^, fresh tumor samples were collected and dissociated into single cells. The tissue was washed with PBS, then chopped and incubated in an enzyme digestion solution for 40 min at 37 °C. After digestion, the solution was filtered through a 40 μm filter and centrifuged. Red blood cells were removed using a red blood cell lysis solution, followed by cell counting. The cell suspension was filtered using FACS tubes, centrifuged again, and washed twice. Finally, after microscopic examination and cell counting, the single-cell suspension was used for scRNA sequencing.

The scRNA sequencing analysis was carried out according to a single cell analysis workflow with BD Rhapsody™ Systems (BD Biosciences, USA), and RNA sequencing and data analysis were completed on the platform of NovaSeq 6000 system (Illumina, USA). The entired scRNA sequencing workflow was provided in the Supplementary document 1.

### Animal model

Six-week-old female BALB/c nude mice and C57BL/6 female mice were used for tumor model establishment. LLC (1 × 10^6^ per mouse) with or without overexpressing CCL28 were subcutaneously injected in C57BL/6 mice (*n* = 6, each group). Mice inoculated with CCL28 knock-out LLC cells and wild-type LLC were randomly divided into two groups, respectively, and treated with or without RA by gavage daily (n = 6, each group). Mice were observed, and the tumors' length and width were measured daily. When tumors grew to a specific size, subcutaneous tumors were collected and photographed. The A549 (CCL28 overexpression or CCL28 knock-out) tumor model followed the same procedure. All animal experiments were carried out following the institutional guidelines and approved by the Ethical Review Committee of Comparative Medicine, Jinling Hospital, Nanjing, China (2022DZGKDWLS-0091).

### Western blot and ELISA

Proteins were extracted from the cultured cells by lysis buffer, separated by SDS-PAGE, and transferred to PVDF membranes (Millipore, USA). The filters were blocked in Tris-buffered saline containing 0.2% Tween plus 5% non-fat milk and incubated with primary antibodies overnight at 4 °C. Secondary antibodies were used for visualization through chemiluminescence (ECL, Amersham Pharmacia Biotech, UK). Primary antibodies against RXRα (CST,1:1000), RARα (CST,1:1000), RDH13 (Proteintech,1:1000), DHRS11 (Proteintech,1:1000), CCR10 (Abmart,1:1000), β-actin (Servicebio, 1:1000) and neutralizing antibody against CCR3 (R&D Systems, USA) were applied in the present study. Anti-rabbit IgG, HRP-linked Antibody (CST, 1:1000) and anti-mouse IgG, HRP-linked Antibody (CST, 1:1000) were utilized as the secondary antibodies. CCL28 in serum was detected by an ELISA kit (Abcam, USA) according to the manufacturer's instructions.

### Immunofluorescence and multiplex Immunohistochemistry (mIHC)

Tumor samples were collected and fixed in 10% formalin before processing and paraffin embedding. Immunofluorescence was conducted on 5 µm sections. For culture cell staining, lung adenocarcinoma-associated pericytes were washed with 1 × PBS and fixed with acetone for 10 min on ice. Tumor microvascular endothelial cells and cancer pericytes were stained by CD31 antibody (Abcam, ab281583) and rat anti-human/mouse monoclonal NG2 antibody (Abcam, ab129051). At the same time, tumor cells were stained by mouse anti-human pan-cytokeratin (Pan-ck) monoclonal antibody (Abcam, ab215838). Goat anti-mouse IgG antibody (labeled with Alex Fluor 488), goat anti-rabbit IgG antibody (labeled with Alex Fluor 555 or Alex Fluor 488), and goat anti-rat IgG antibody (labeled with Alex Fluor 555 or Alex Fluor 488) (Abcam) were applied as secondary antibody in immunofluorescence staining analysis.

For multiplex immunohistochemistry, the slides were incubated with the primary antibodies (anti-CD31, anti-NG2, anti-Pan-CK antibody, and anti-CCL28 (Proteintech, 18,214–1-AP), respectively) and horseradish peroxidase-conjugated secondary antibody, and tyramine signal amplification (TSA) was performed following the pre-optimized antibody concentration and the order of staining. Antibody stripping and antigen retrieval were performed after each round of TSA. DAPI (Sigma-Aldrich, USA) was used for nuclei staining. A whole slide scan of the multiplex tissue sections produced multispectral fluorescent images visualized in SlideViewer software. A specialized pathologist chose representative regions of interest (ROI), and multiple fields of view were acquired at 20 × power for further analysis. The mean fluorescence intensity of CCL28 in each ROI was calculated by the ImageJ program (https://imagej.nih.gov/ij/).

### CRISPR-cas9 knock out

Briefly, three designed sgRNAs were synthesized and co-transfected with Cas9 nuclease/sgRNA in A549 lung adenocarcinoma cells. After 72 h, the cells were seeded in a 96-well plate for single-cell cloning by limiting dilution analysis (LDA). After 7 to 15 days, about ten single-cell clones were selected for PCR and ELISA. Mutant sites were further confirmed by DNA sequencing.

### Knockdown of RDH13 and DHRS11

For RDH13 and DHRS11 gene silence, pericytes were infected with negative control siRNAs, RDH13-targeting, or DHRS11-targeting siRNAs (genepharma). Briefly, pericytes were seeded in 24-well plates, and the cells were transfected with siRNAs using Lipofectamine RNAiMAX (Invitrogen) according to the manufacturer’s instructions.

### Bioinformatic analysis

Transcription factor binding sites prediction in promotor of CCL28 gene was conducted in PROMO 3.0, virtual laboratory TFSEARCH (ver.1.3) and JASPAR (https://alggen.lsi.upc.edu/rerecer/menu_recerca.html, https://diyhpl.us/~bryan/irc/protocol-online/protocol-cache/TFSEARCH.html, https://jaspar.elixir.no/). RNA sequencing dates of lung adenocarcinoma tumor samples or lung adenocarcinoma cell lines were extracted on the cBioPortal platform (https://www.cbioportal.org/). The relationship between expression levels of CEBPB and CCL28, RXRα, and ANNGPT1 were calculated online, as well as the predictive value of CEBPB for the survival of lung adenocarcinoma patients.

### Statistical analyses

Data were presented as mean ± SEM. Student's unpaired two-tailed tests were used for comparisons between two groups. Pearson or Spearman correlation was applied to analyze the relationship between the expression scores of two genes. Statistical analyses were performed on GraphPad Prism 8.0. Multiple comparisons were analyzed by one-way ANOVA using the LSD test. Statistical significance was confirmed when *p* < 0.05.

## Results

### CCL28 expression is up-regulated after anti-angiogenesis therapy by hypoxia-sensitive transcription factor CEBPB in lung adenocarcinoma

The GEO database (https://www.ncbi.nlm.nih.gov/gds/) was retrieved to elucidate the impact of anti-angiogenesis therapy on the gene expression of cancer patients. Molecular expression profile data (GSE61676) of lung adenocarcinoma patients undergoing anti-angiogenesis treatment was reanalyzed. Twenty-three stage IV lung adenocarcinoma patients, treated with a combination of anti-VEGF monoclonal antibody (Bevacizumab) and TKI, were selected for analysis. The changes in mRNA profile (Affymetrix Human Exon 1.0 ST Arrays) were examined before and 24 h after Bevacizumab treatment. The findings revealed a significant increase in the hypoxia index (GLUT1, one of the primary target genes regulated by hypoxia-inducing factor HIF-1) and up-regulation of CCL28 expression in lung adenocarcinoma patients 24 h after VEGF monoclonal antibody treatment compared to baseline levels (Fig. 1A, 1B). After treatment with bevacizumab, CCL28 expression showed a log2 fold change of 1.258 compared to baseline, with a corresponding p-value of 0.0152, as depicted in Fig. 1A. After anti-angiogenesis therapy, tumor cells often experience a state of ischemia and hypoxia. To simulate this hypoxic state of tumor cells, nine cell lines of 4 different tumor types were culture in hypoxia and normoxia conditions, including two hepatoma cell lines (HEPG2 and SMMC), three lung adenocarcinoma cell lines (A549, SPC-A1, and H1975), two breast carcinoma cell lines (MCF7 and MDA-MB-231), and two colorectal cancer cell lines (HCT116 and SW480). The expression of GLUT1 and VEGFA, which was driven by the hypoxia-induced factor (HIF), was detected by qRT-PCR. VEGFA and GLUT1 were up-regulated in all nine cell lines in the hypoxic chamber, indicating that the hypoxic microenvironment was established (Fig. 1C). We previously reported that high expression of CCL28 was induced in hypoxic lung adenocarcinoma cell lines, A549 and SPC-A1. To further confirm our study, we examined the expression of CCL28 under hypoxia conditions in the nine cell lines. CCL28 was up-regulated in all three lung adenocarcinoma cell lines, whereas others were not (Fig. 1D). Then, we utilized PROMO 3.0 to predict the transcription factors binding sites to the CCL28 promoter region and intersected them with previously reported hypoxia-related transcription factors, revealing six transcription factors involved (Fig. 1E). CEBPB and Sp1 can potentially regulate CCL28, while only CEBPB was highly expressed in hypoxic tumor cells (as listed in Supplementary Table 1). We then used a public integrative database (cBioPortal) to analyze the correlation of the expression of CEBPB with that of CCL28 in 44 lung adenocarcinoma cell lines (as listed in Supplementary Table 2). As expected, CEBPB expression strongly correlated with CCL28 (Fig. 1F). The CEBPB binding motif was shown in Fig. 1G. Thus, we speculated that hypoxia-induced high expression of CCL28 was mediated by CEBPB. To verify our prediction, we generated luciferase reporter plasmids harboring either wild-type (WT) or mutated (MUT) CEBPB binding sequencing within the promotor element of the *CCL28* gene. Luciferase reporter analysis indicated that CEBPB could directly regulate the expression of the *CCL28* gene in A549 cells (Fig. 1H). In addition, disease-free survival and overall survival of lung adenocarcinoma patients with high CEBPB expression were significantly decreased (*p* = 0.0065 and *p* = 0.032, respectively) (Fig. 1I).

### Tumor-derived CCL28 promotes vascular normalization and pericyte recruitment in the tumor microenvironment

**Fig. 2:**
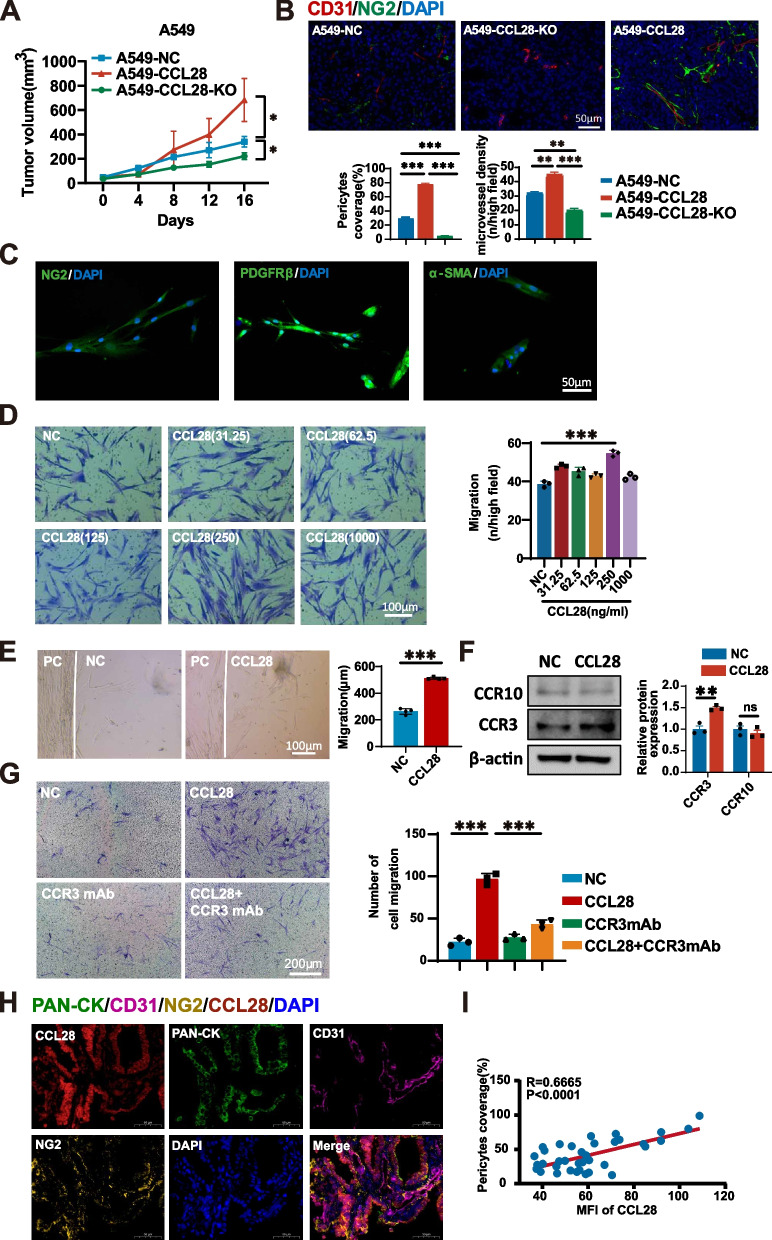
Tumor-derived CCL28 recruits pericytes to promote vascular normalization in the tumor microenvironment

To investigate the effects of CCL28 on tumor growth and vascular normalization, we established CCL28 overexpression and knock-out lung adenocarcinoma cells by lentivirus vectors and Cas9 nuclease/sgRNA (Supplementary Fig. 1), respectively. As we previously reported, in vivo studies indicated that CCL28 could promote tumor growth in A549 human lung adenocarcinoma (Fig. 2A). Microvessels and pericytes were subsequently assessed by immunofluorescence staining using antibodies against CD31 (red) and NG2 (green) in tumor tissue in different groups. The percentage of pericyte coverage and microvessel density (MVD) in A549-CCL28 tumor tissue significantly increased compared to A549-NC, and this effect was reversed after *CCL28* was knocked out (Fig. 2B). These results suggested that CCL28 could promote vascular normalization and mobilize pericytes.

Further, we isolated primary pericytes from lung cancer tissues to explore how CCL28 recruits pericytes in vitro. The characteristics of pericytes were identified by immunofluorescence staining α-SMA, PDGFRβ, and NG2 (Fig. 2C). Cell viability remained unaffected by different concentrations of recombinant CCL28 in pericytes and A549 (Supplementary Fig. 2A,2B), but enhanced cell migration of A549 (Supplementary Fig. 2C). The transwell migration assay was performed to confirm the effect of CCL28 on the migration of pericytes by using different concentrations of human recombinant CCL28 protein (Fig. 2D, left panel). The number of cells migrated to the lower chamber was calculated (Fig. 2D, right panel), and we found that the optimal concentration of CCL28 for enhancing the migration efficiency of pericytes is 250 ng/ml. Thus, we selected a concentration of 250 ng/ml to conduct subsequent migration experiments. To further verify the recruitment effect of CCL28 on pericytes, cells were inoculated around matrigel mixed with or without CCL28 recombinant protein. The graphs visually illustrate cell migration distance and quantity (Fig. 2E, left panel). CCL28 significantly increased the migration distance of pericytes compared to the control group (Fig. 2E, right panel). Subsequently, we assessed the expression of two CCL28 receptors, CCR3 and CCR10, in pericytes with or without CCL28. Western blot experiments revealed that the expression of CCR3 was significantly higher than that of CCR10 in pericytes, suggesting that CCR3 likely plays a predominant role in mediating CCL28 signal transduction within pericytes (Fig. 2F). Blocking CCR3 with neutralizing antibodies diminished the chemotactic effect of CCL28 on pericytes (Fig. 2G). Further, we validated the relationship between CCL28 and pericytes coverage in biopsy tissue using multiplex immunofluorescence. CCL28 was mainly expressed in lung adenocarcinoma cells due to the fluorescence overlapping between CCL28 and PAN-CK (Fig. 2H). We found a significant positive correlation between CCL28 expression levels and the percent of pericytes coverage (Fig. 2I). The above findings indicate that CCL28 could recruit pericytes through the receptor CCR3, thereby promoting vascular normalization. Because the action of CCL28 leads to vascular normalization, making the vascular network healthier and more regular, it may also increase the density and functionality of tumor blood vessels, providing more nutrients and oxygen to promote tumor growth.

### Tumor-derived CCL28 promotes expression of angiopoietin-1 via CCR3 in pericytes

**Fig. 3:**
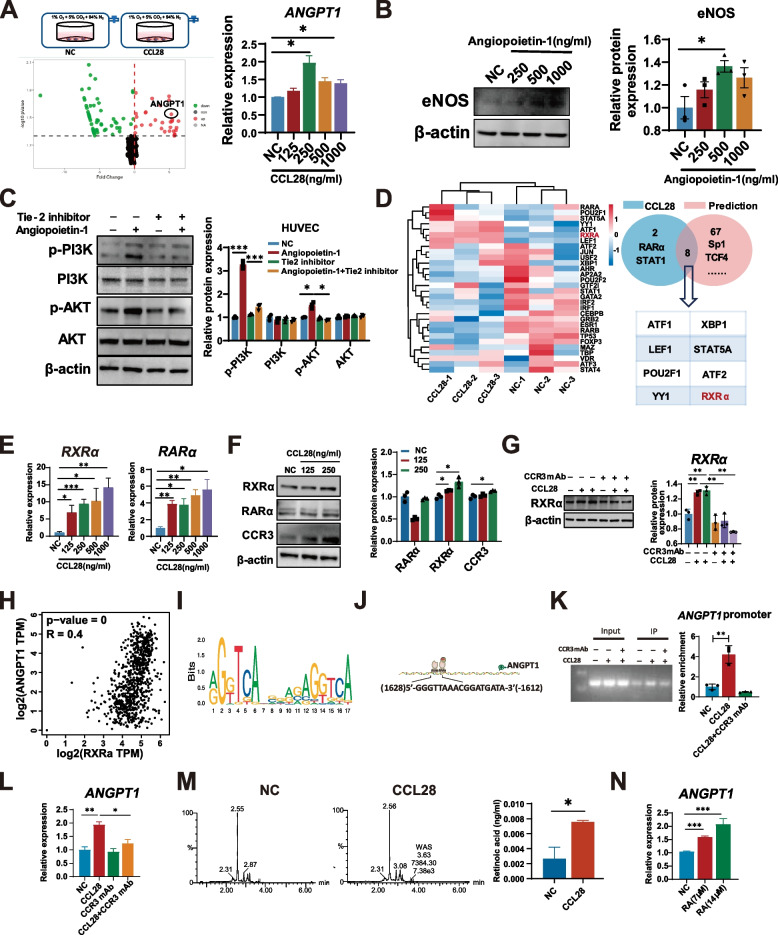
Tumor-derived CCL28 promotes the expression of angiopoietin-1 via CCR3 in pericytes

Pericytes interact with vascular endothelial cells to promote the maturation of neovasculature [25]. However, the molecular mechanisms underlying pericytes-induced vascular normalization in lung adenocarcinoma after anti-angiogenesis therapy remain unclear. To clarify the exact role and potential mechanism of CCL28 in vascular normalization, we mimicked the hypoxic microenvironment in lung adenocarcinoma, cultured vascular pericytes, and conducted secretome analysis after CCL28 treatment. We detected augmented production of angiopoietin-1 in pericytes stimulated by CCL28 (Fig. 3A). Compared to the control group, the expression of ANGPT1 changed by 5.415-fold under CCL28 stimulation, with a p-value of 0.033. RNA-seq results were confirmed by quantitative PCR in different concentrations of recombinant CCL28 protein under hypoxia conditions (Fig. 3A right panel). Angiopoietin-1, a protein typically associated with angiogenesis and vascular normalization, maintains vascular stability and integrity by interacting with endothelial cells. Consistent with prior studies [24, 26], western blot experiments showed that recombinant human angiopoietin-1 could up-regulate endothelial nitric oxide synthase (eNOS) expression in a dose-dependent manner in human umbilical vein endothelial cell (HUVEC) (Fig. 3B). Likewise, recombinant human angiopoietin-1promoted cell survival by activating the PI3K-AKT signaling pathway in a Tie2-dependent manner in endothelial cells (Fig. 3C). To further explore the regulatory mechanism of ANGPT1 expression, we identified a significant increase in the transcription factor RXRa and RARα after CCL28 stimulation in transcriptome data, verified by qPCR (Fig. 3D and E). However, protein level detected by western blot, CCL28 up-regulated RXRα and CCR3 but not RARα (Fig. 3F). Also, the effect of CCL28 on RXRα up-regulation was reversed by adding a CCR3 neutralizing antibody (Fig. 3G). Next, we confirmed the relation between transcription factor RXRα and expression of ANGPT1. Correlation analysis showed that RXRα expression positively correlates with ANGPT1 expression (Fig. 3H). The binding motif of the RARα and RXRα complex (Fig. 3I) and the transcription factor binding sites in the promotor area of the CCL28 gene (Fig. 3J) were shown. CHIP-qPCR was performed to investigate the binding of transcription factor RXRα to the promoter region of the target gene *ANGPT1*. Compared to the NC group, CCL28 enhanced the degree of enrichment, and this effect could be dampened after the CCR3 neutralization, suggesting a direct interaction between RXRα and the ANGPT1 promoter. The electrophoresis image displayed the specificity of the amplified DNA fragments (Fig. 3K).

Furthermore, the mRNA level of ANGPT1 was increased by the addition of CCL28 but attenuated by the blockade of CCR3 (Fig. 3L). RXRα is a subtype of nuclear receptor closely associated with vitamin A metabolism and retinoic acid signaling. These results led us to speculate whether retinoic acid metabolism was also involved in regulating vascular normalization by CCL28. Thus, we measured the retinoic acid content in pericytes using LC–MS technology. Interestingly, the retinoic acid production was notably increased (almost 2.5-fold change) compared to the control group in pericytes stimulated by CCL28 (Fig. 3M). Additionally, the exogenous addition of retinoic acid in pericytes could promote the expression of ANGPT1 in a dose-dependent manner (Fig. 3N). These results indicate that CCL28 can activate the nuclear transcription factor RXRα through the CCR3 receptor, promoting the expression of ANGPT1 in pericytes. In interaction with endothelial cells, pericytes-deriore, the mRNA level of ANGPT1 was increased by the addition of CCL28 but attenuated by the blockade ofon.

### CCL28 activates retinoic acid signaling in pericytes through CCR3

**Fig. 4:**
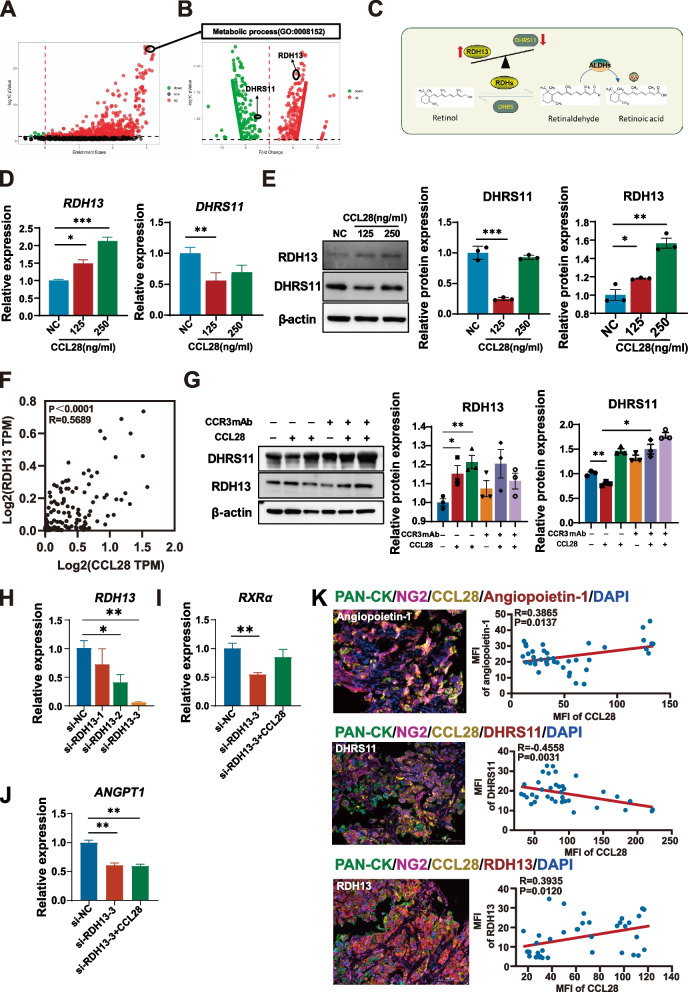
Retinoic acid signaling is activated by CCL28 in pericytes through CCR3** A**, Volcano plot of changes in metabolic pathways after CCL28 stimulation. **B** Volcano plot of the enrichment of gene expression after CCL28 stimulation. **C** Diagram of the metabolic conversion process in the retinoic acid metabolic signaling pathway. **D** and **E** Expression of RDH13 and DHRS11 detected by qPCR and western blot with or without exogenous supplement of CCL28. **F** Correlation of expression of CCL28 with RDH13 in lung adenocarcinoma. **G** The protein level of DHRS11 and RDH13 stimulated with or without CCL28 and CCR3 neutralizing antibody in pericytes (left) and gray value was calculated(right). **H** Knockdown efficiency of RDH13 was confirmed by qPCR. **I** and **J** Relative expression of RXRα and ANGPT1 after knockdown of RDH13 with or without stimulation of CCL28. **K** Representative immunofluorescence images of PAN-CK, NG2, CCL28 with DHRS11 or RDH13 or Angiopoietin-1 on biopsy tissues from lung cancer patients (left panel). Scale bar = 100 μm. The correlation between the expression of CCL28 and the levels of DHRS11, RDH13, and angiopoietin-1 (right panel). Data with error bars are shown as mean ± SEM. Each symbol represents data from a replicate. Each panel is a representative experiment of at least three independent biological replicates. *, **, *** represent *p* < 0.05, *p* < 0.01 and *p* < 0.001, respectively. Abbreviation: MFI, Mean fluorescence intensity

We further analyzed the sequencing data to understand better the mechanism responsible for retinoic acid accumulation in pericytes after CCL28 stimulation. Enrichment analysis of gene pathways showed that RA signaling pathways were activated after CCL28 stimulation (Fig. 4A). Two key enzymes involved in retinoic acid metabolism have changed, including RDH13 and DHRS11 (Fig.4B). RDH13 plays a role in converting retinol to retinaldehyde, a critical rate-limiting enzyme step in the retinoid metabolic pathway. However, DHRS11 reduces retinoic acid metabolites to related forms of retinol. In this way, the balance between RDH13 and DHRS11 can affect the level of retinoic acid in cells. The metabolic balance model diagram of retinoic acid shows the transformation process of the three substances (Fig. 4C). In this metabolic process, RDH13 was up-regulated, but DHRS11 was decreased in pericytes after treatment of CCL28 (Fig. 4B). These results were confirmed by qPCR (Fig. 4D) and western blot (Fig. 4E). The correlation analysis exhibited a positive relationship between RDH13 and CCL28 in lung adenocarcinoma (Fig. 4F). Expression changes of RDH13 and DHRS11 in pericytes stimulated by CCL28 could be reversed by blocking CCR3 (Fig. 4G). However, treating pericytes directly with retinoic acid did not result in changes in key molecules involved in RA metabolism (Supplementary Fig. 3A). To further investigate whether the expression of two key enzymes in the retinoic acid synthesis process is correlated with the expression of ANGPT1, we knocked down the expression of RDH13 and DHRS11 using siRNA and then examined the expression of ANGPT1. After knockdown of RDH13 (Fig. 4H), RXRα and ANGPT1 were significantly reduced (Fig. 4I, J). However, DHRS11 did not exhibit this effect (Supplementary Fig. 3B, 3C). To further elucidate the relationship between CCL28 and its downstream molecules, immunofluorescence staining of key molecules—RDH13, DHRS11, and ANGPT1—was performed on tumor biopsy tissues (Fig. 4K, left panel), followed by correlation analysis. In these samples, we observed a positive correlation between CCL28 expression and the levels of ANGPT1 and RDH13, while a negative correlation was found with DHRS11 (Fig. 4K, right panel).All these results illuminated that CCL28 promoted the retinoic acid synthesis process by disturbing the balance between RDHs and DHRS, but the changes are temporary and dynamic.

### Both CCL28 and retinoic acid could promote vascular normalization in vivo

**Fig. 5:**
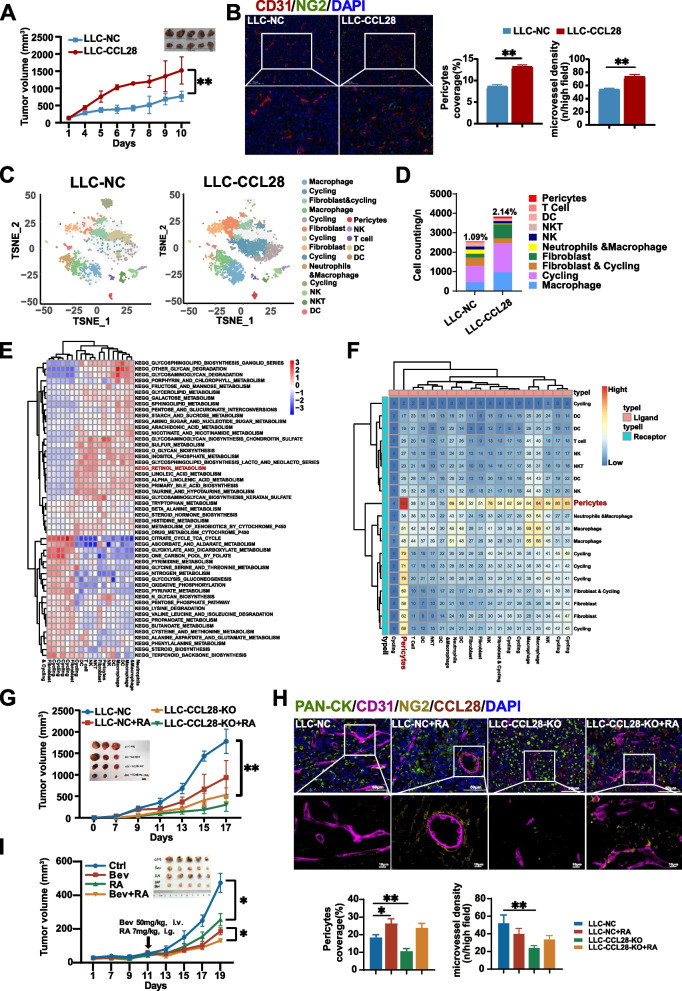
Both CCL28 and retinoic acid could promote vascular normalization in vivo

Further, we investigated the role of CCL28 in tumor growth and vascular normalization in immunocompetent mice. We overexpressed CCL28 in LLC cells, which was validated by ELISA. Compared to the control group, CCL28 overexpression showed a significant increase in CCL28 concentration in both the cell supernatants and serum in mice implanted with tumors (Supplementary Fig. 4A, 4B). As expected, in vivo studies indicated that CCL28 could promote tumor growth in Lewis lung adenocarcinoma (LLC) (Fig. 5A). Compared to the control group, NG2 + pericytes accumulation and angiogenesis enhanced in the LLC-CCL28 group (Fig. 5B).

To more clearly explore the function of CCL28 on stromal cells and immune cells in the tumor microenvironment, we performed single-cell sequencing analysis on tumor tissues of LLC-NC and LLC-CCL28. Single-cell sequencing analysis provided a panoramic study of the tumor microenvironment, and the cell population in the tumor microenvironment underwent significant alterations. In total, 6380 cells were obtained after quality filtering in the 2 conditions. Specifically, there were 3829 cells in the CCL28 overexpression group, and 2551 cells in the control group. The t-SNE diagrams showed that these cells were divided into 19 cell clusters with a total of 10 cell types (Fig. 5C and Supplementary Fig. 4D, 4E). Of all the cell types, three types showed the most significant changes in cell proportions: fibroblasts, pericytes, and macrophages, with fold changes of 2.39, 1.95, and 1.45, respectively. The percentage of pericytes was 1.09% in LLC-NC and rose to the rate of 2.14% in LLC-CCL28 (Fig. 5D). In the CCL28 overexpressing group, we identified 82 pericytes, whereas the control group had 28 pericytes. This significant difference suggested that CCL28 may play a pivotal role in influencing pericyte populations within the tumor microenvironment. Moreover, a previous study has reported that CCL28 could recruit Tregs and promote angiogenesis in ovarian carcinoma [17]. However, the percentage of T cells was reduced in the CCL28 up-regulated LLC, indicating that CCL28 might play different roles in different cancer types. Moreover, the number of DC cells remains unchanged, but their proportion decreased (Fig. 5D).

Subsequently, we focused on the analysis of pericytes. Cluster 14, which was CSPG4^+^ and ACTA2^+^, was recognized as pericytes. The marker genes t-SNE plot for Cluster 14 was presented in Supplementary Fig. 5. Interestingly, metabolic pathway enrichment analysis revealed that the retinol metabolism was activated in 13 clusters, including DC, macrophages, fibroblasts, and pericytes, suggesting that the synthesis and metabolism of retinoic acid play essential physiological roles in tumor microenvironment (Fig. 5E). In the cellular communication network, pericytes interact closely with various cell types. The heat map indicated that pericytes exhibited the strongest interaction with themselves, followed by cluster 3 and cluster 6, representing tumor and endothelial cells. Cluster 3 was characterized as Ki-67-positive and Top2a-positive, and cluster 6 was positive for Pecam (Fig. 5F). Our results in Fig. 3M also suggested CCL28 can promote retinoic acid accumulation in pericytes in vitro. We further investigated the effects of retinoic acid by daily gavage on tumor growth and vascular normalization in vivo. Knocking out CCL28 (LLC-CCL28-KO) or supplementing retinoic acid (LLC-NC + RA) could suppress tumor growth in LLC models (Fig. 5G and Supplementary Fig. 4C). However, the suppression effect is more pronounced after knocking out CCL28 than the control group (LLC-NC), and CCL28 knockout with supplementing RA could further synergistically inhibit tumor growth (Fig. 5G). Multiplex immunofluorescence was used to detect the normalization of tumor blood vessels. We found that compared to the control group, knocking out CCL28 resulted in a significant reduction in the proportion of NG2 + cells and decreased coverage of pericytes. As expected, supplementation of retinoic acid was able to promote a certain degree of restoration of pericytes coverage both in wild-type and CCL28 knockout tumors (Fig. 5H). Then, we quantified the vascular density in different groups and found that, compared to the control group, CCL28 knockout resulted in significantly decreased vascular density, while retinoic acid had only a slight effect (Fig. 5H). Additionally, we evaluated the effect of retinoic acid on tumor cell viability using a CCK8 assay (Supplementary Fig. 4F). The results indicated that retinoic acid did not significantly affect tumor cell viability at pharmacological concentrations (1–2 μg/ml, equivalent to 3.33–6.66 μM). However, cytotoxic effects were observed at higher concentrations, which exceed pharmacologically relevant levels in the body necessary to support essential cellular functions. To confirm the influence on tumor hypoxia, we immunofluorescently labeled mouse tumor tissues using CA9 (Carbonic Anhydrase 9), which is considered an indicator of hypoxia. Our findings indicated that compared to the control group, tumor hypoxia significantly increased in tumors with CCL28 knockout. However, retinoic acid does not appear to have a significant effect on hypoxia (Supplementary Fig. 4G). These results indicated that RA inhibited tumor growth and enhanced vascular normalization, rather than angiogenesis.

The above data indicate that CCL28-regulated retinoic acid plays a crucial role in vascular normalization, suggesting its potential as a therapeutic agent in anti-angiogenic tumor treatment. To investigate whether the combination of retinoic acid and bevacizumab has a synergistic effect, we established an A549 mouse model and administered intravenous injections of bevacizumab, coupled with oral gavage of retinoic acid. We observed that retinoic acid and bevacizumab could attenuate tumor growth in mice, respectively. Moreover, simultaneous administration of retinoic acid and bevacizumab had a synergistic effect on tumor growth (Fig. 5I). These data indicate that retinoic acid can be used in combination with bevacizumab for tumor treatment, providing a new direction for clinical combination therapy.

### CCL28 is involved in bevacizumab-mediated vascular normalization

**Fig. 6:**
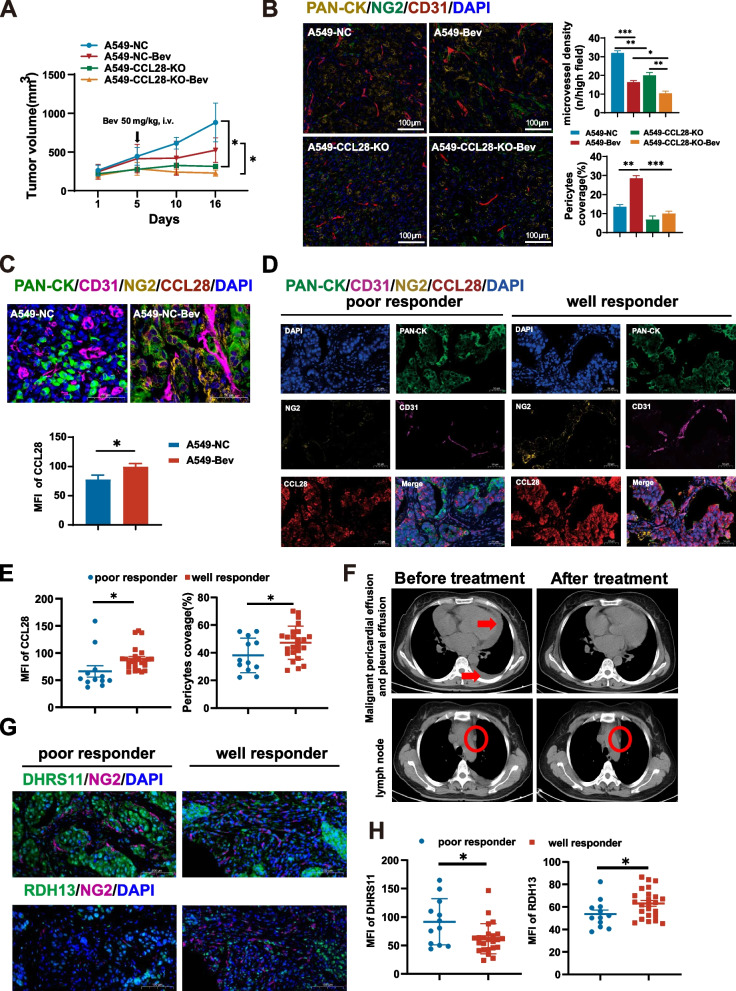
CCL28 is involved in bevacizumab-mediated vascular normalization

Moreover, we investigated the synergistic effects of CCL28 knock-out and VEGF blocking on the tumor growth and vascular normalization of lung adenocarcinoma. Subcutaneously implanted lung adenocarcinoma cells with CCL28 knock-out grew much slower than wild-type tumor cells, while combination of VEGF blocker could stop the growth of the tumors (Fig. 6A). In addition, significant promotion of vascular normalization is observed after bevacizumab treatment. However, this effect disappeared after knocking out CCL28, regardless of whether bevacizumab was added (Fig. 6B). Knocking out CCL28 could inhibit tumor angiogenesis, and the effect was more pronounced when bevacizumab was combined (Fig. 6B). These results indicated that CCL28 could participate in bevacizumab-mediated vascular normalization, and CCL28 might be a potential target for anti-angiogenesis therapy in lung adenocarcinoma.

Further, we evaluated the expression levels of CCL28 within the tumor tissue (Fig. 6C, upper panel). The mean fluorescence intensity of CCL28 was significantly upregulated when bevacizumab was used compared to the control group (Fig. 6C, lower panel), consistent with the results we found in the clinical samples. Also, the expression level of CCL28 in lung adenocarcinoma correlates with therapeutic efficacy. In lung adenocarcinoma patients, based on their treatment outcomes with bevacizumab, they were categorized into two groups: poor responders and good responders. PAN-CK, NG2, CD31, and CCL28 were stained in tumor tissues to analyze their expression levels with the therapeutic efficacy of bevacizumab treatment. Patients who responded well to bevacizumab showed significantly increased CCL28 expression, and high expression of CCL28 in tumor cells,, was associated with enhanced vascular maturity and favorable treatment outcomes (Fig. 6D, 6E). The conclusion was evidenced by a notable decrease in malignant pericardial and pleural effusion and a significant reduction in metastatic lymph nodes after bevacizumab-based treatment (Fig. 6F). Then, we analyzed the expression levels of DHRS11 and RDH13 in two groups of patients. Consistently, the therapeutic efficacy of bevacizumab appeared to be positively correlated with RDH13 expression and negatively correlated with DHRS11 expression (Fig. 6G, H). These results suggest that the favorable response to anti-angiogenic therapy in patients may be attributed to the activation of CCL28, thereby deepening our understanding of treatment response variations.

## Discussion

Anti-angiogenesis therapy stands as a pivotal approach for metastatic lung adenocarcinoma [3, 27], targeting the well-established VEGF/VEGFR pathway with numerous drugs developed over the last four decades [28, 29]. However, its clinical impact has proven more complicated than initially anticipated. Recognizing the vasculature normalization effects of anti-angiogenic drugs has led to their integration into combination regimens with chemotherapy, radiotherapy, immunotherapy, and targeted therapy [4]. For example, bevacizumab, through the modulation of angiogenesis and improvement of the tumor microenvironment, lowers tumor vascular density and edema, thereby enhancing the efficiency of oxygen supply and making other treatment modalities more effective. Consequently, anti-angiogenesis drugs are employed as modulators for tumor vasculature and the microenvironment within combination regimens.

Anti-angiogenic therapy inhibits abnormal blood vessel formation, fostering vascular normalization through mechanisms like reducing vessel density, remodeling the extracellular matrix, regulating inflammation, balancing growth factors, and modulating the immune system. The tipped balance between pro-angiogenesis and anti-angiogenesis factors contributes to maintaining tumor vascular growth [30]. Anti-angiogenic therapy disrupts this tipped balance by inhibiting VEGF, enhancing the relative action of angiopoietin-1(ANGPT1), which aids in regulating and inducing vascular normalization. This rebalancing contributes to vascular normalization, ensuring newly formed blood vessels exhibit a more organized structure and function more normally. The action of angiopoietin-1 helps consolidate and stabilize the newly formed vessels, making them a more effective transportation system.

However, it is also widely accepted that agents targeting VEGF/VEGFR may ultimately elevate hypoxia levels within tumors [4]. This hypoxic microenvironment triggers alternative pro-angiogenesis molecular pathways in tumor or stromal cells within the tumor microenvironment, including FGF-2, HGF, DLL4/Notch, CCL28, and others [19, 21, 22, 31]. Furthermore, hypoxia prompts a cascade of biological responses, leading tumor cells to adapt their metabolic pathways to low-oxygen conditions. The dysregulated metabolism observed in rapidly proliferating tumor cells is a hallmark of malignancy [32], contributing to the activation of various metabolism-related genes, including several hypoxia-related transcription factors like hypoxia-inducible factor (HIF), nuclear factor kappa-B, CREB, AP-1, p53, Sp1/3, Egr-1, and CEBPB [33].

In this study, we identified CCL28 as another crucial molecular target for vascular normalization in lung adenocarcinoma. It was observed that CCL28 could be up-regulated under hypoxic conditions. However, the molecular mechanism underlying hypoxia-induced transcriptional activation of the CCL28 gene remains unclear. Our investigation revealed that the transcription factors CEBEPB could regulate the expression of CCL28. CCL28, belonging to the subfamily of small cytokine CC chemokines, binds to chemokine receptors CCR3 and CCR10 [19]. It has been reported that CCL28 exhibits chemotactic activity for various immune cells and plays a role in the physiology of extracutaneous epithelial tissues [34]. Several studies have delved into the functions of CCL28 in the tumor microenvironment [17, 18, 20]. Therefore, we hypothesized that CCL28 might play a pivotal role in modulating the tumor microenvironment in lung adenocarcinoma. Here, we noted an augmentation in pericyte accumulation within the tumor microenvironment associated with the overexpression of CCL28. However, the mechanisms of pericyte recruitment in lung adenocarcinoma remain unknown. Thus, we examined the expression of CCR3/CCR10 on various tumor stromal cells, revealing widespread expression of CCR3 on endothelial cells, cancer-associated fibroblasts, and pericytes in lung adenocarcinoma. Two chemotactic experiments substantiated the effects of CCL28 on recruiting pericytes through the receptor CCR3.

Abnormal differentiation of stromal cells is an essential characteristic of malignant tumors and is correlated with abnormal metabolism. Many tumor stromal cells were differentiated from anti-tumor type to pro-tumor type, such as tumor-associated macrophage, cancer-associated fibroblasts, tumor-associated neutrophils, etc. [35–37]. Reprogramming the metabolism and modulating tumor stromal cell differentiation is a promising cancer treatment strategy. Interestingly, the present study found that retinoic acid metabolism was activated in a series of stromal cells in lung cancer.

Importantly, chemotactic factors play a crucial role in cell migration, influencing not only the cell's movement [38] but also closely interacting with cellular metabolism. They regulate energy production, metabolic pathways, and antioxidant responses, ensuring that cells have sufficient energy and resources during migration. Chemotactic factors influence cellular metabolism by regulating intracellular signaling pathways, such as PI3K/AKT, MAPK, AMPK, and mTOR [39, 40]. The activation or inhibition of these signaling pathways can modulate the activity of intracellular metabolic enzymes, affecting processes such as glucose metabolism, lipid synthesis, and amino acid utilization. In the present study, we found chemokine CCL28 could influence the cellular levels of retinoic acid by modulating its synthesis.

Retinoic acid (RA), also known as vitamin A acid, and its related analogs participate in regulating the gene networks involved in cell growth, differentiation, homeostasis, and apoptosis. RA is an active metabolite of retinol. Retinol (Vitamin A) is a fat-soluble essential micronutrient that plays a crucial role in embryonic development, organ formation, immune system function, and vision [26]. Retinol can be metabolized into retinal by the retinol dehydrogenases (RDHs), and retinal can also be metabolized into retinol by the dehydrogenase/reductase SDR family (DHRS) [41]. Retinal can be further irreversibly metabolized to RA by the retinaldehyde dehydrogenases (ALDHs). The synthesis of RA depends on the balance between RDHs and DHRS. The present study found that CCL28 could disturb the balance between RDH13 and DHRS11. After being treated with CCL28, RDH13 in pericytes was significantly upregulated in a dose-dependent manner. However, the effect curve of CCL28 on DHRS11 expression in pericytes is not linear and might be bell-shaped, like the effects of VEGFA on endothelial cells. In addition, there might be an alternative pathway to maintain the balance of DHRS11 and RDH13 in pericytes. RA binds to RARα, promoting the formation of a heterodimer with retinoid X receptor alpha (RARα/RXRα) in the cell nucleus. This heterodimer binds to retinoic acid response elements (RAREs) in the promoter region of target genes [42], including *ANGPT1*.

The angiopoietin family, including ANGPT1, ANGPT2, ANGPT3, and ANGPT4, is crucial in vascular development and normalization. They regulate endothelial cells' survival, proliferation, and migration by interacting with Tie receptors, thus vital to vascular normalization. ANGPT1 can activate the TIE2 receptor on endothelial cells, maintaining endothelial cell stability, enhancing tight connections between endothelial cells, and reducing microvascular permeability through a series of signaling pathways [26]. These signaling pathways include tyrosine kinase-related protein DOKR (also known as DOK2), endothelial nitric oxide synthase (eNOS), SH2 domain-containing phosphatase (SHP2), growth factor receptor-binding protein 2 (GRB2), and PI3K-Akt [24]. Various regulatory mechanisms influence the expression of the *ANGPT1*. These include the hypoxia-inducible factor-1α (HIF-1α) signaling pathway under low oxygen conditions, interaction with vascular endothelial growth factor (VEGF), involvement of anti-inflammatory factors and growth factors, cell–cell interactions, as well as the nuclear factor-κB (NF-κB) signaling pathway and hormonal regulation. In the present study, we identified another regulatory mechanism controlling the expression of ANGPT1.

Vitamin A and its metabolites, particularly retinoic acid, exert regulatory effects on the vascular system through various pathways [43]. These include maintaining endothelial cell function, regulating angiogenesis, inhibiting vascular smooth muscle cell proliferation, suppressing inflammatory responses, and promoting cell differentiation. This comprehensive regulatory process contributes to maintaining blood vessels' stable structure and function, playing a crucial role in embryonic development, tissue repair, angiogenesis, and vascular health during inflammation and diseases. Interestingly, vitamin A and its metabolites have been used as a differentiation modulator of malignant cells for cancer treatment. The present study proposed a new strategy to modulate the differentiation of cancer stromal by vitamin A and its metabolites.

## Conclusions

**Fig. 7:**
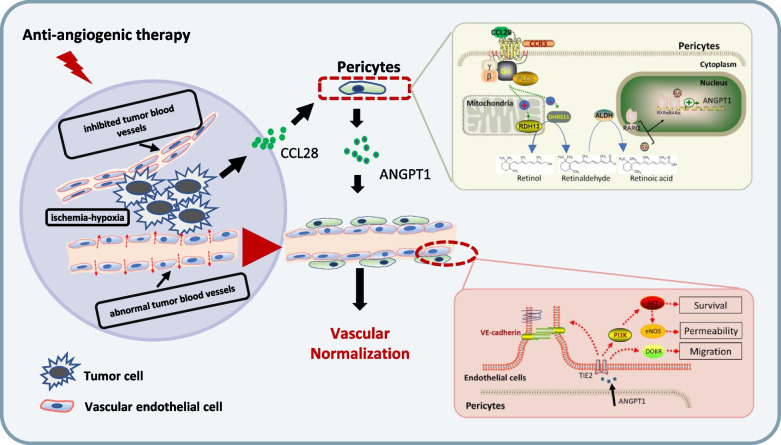
A schematic diagram of tumor microenvironment modulation effects of CCL28

In conclusion, we elucidated that a specific chemokine CCL28 induced after anti-angiogenesis therapy can alter tumor stromal cell metabolism and reshape the tumor microenvironment. In summary, we identified a mechanism through which CCL28 promotes vascular normalization via a CCR3-pericytes-RA-RXRα-ANGPT1-dependent pathway (Fig. 7). With an in-depth exploration of the interplay between chemokine and ANGPT1, our work may provide valuable insights into the regulatory network of vascular normalization, offering new avenues for modulating tumor microenvironment and overcoming resistance to anti-angiogenesis therapy in lung adenocarcinoma treatment.

### Supplementary Information

Supplementary Material 1.

Supplementary Material 2.

Supplementary Material 3.

Supplementary Material 4.

## Data Availability

The data that support the findings of this study are available from the corresponding author upon reasonable request.Competing interests. The authors declare that they have no competing interests.

## Abbreviations

PDGFRβ: Platelet-derived growth factor receptor β
NG2/CSPG4: Chondroitin sulfate proteoglycan 4
CCL28: Human CC motif chemokine ligands 28
HUVECs: Human umbilical vein endothelial cell
DHRS11: Dehydrogenase/reductase 11
RDH13: Retinol dehydrogenase 13
BEV: Bevacizumab
