# Supplementary material for: Pericytes recruited by CCL28 promote vascular normalization after anti-angiogenesis therapy through RA/RXRA/ANGPT1 pathway in lung adenocarcinoma
Source: J Exp Clin Cancer Res. 2024 Jul 29;43:210. doi: 10.1186/s13046-024-03135-3 (PMC11285179; doi:10.1186/s13046-024-03135-3)
Supplement: Supplementary file 1 — Supplementary Material 1. [file 13046_2024_3135_MOESM1_ESM.docx]

**Single-cell RNA sequencing analysis**

The Chromium Controller and the Single Cell Reagent Kit v3.1 (Dual Index) were used to prepare individually barcoded single-cell RNA-Seq libraries following the manufacturer’s protocol (10X Genomics). Cellular suspensions were loaded onto a Single-cell chip together with Single-cell Gel Beads, where gel beads in emulsion (GEM) generation occurred. RNA from the barcoded cells was subsequently reverse-transcribed, and sequencing libraries were constructed using reagents from the Chromium Single Cell Reagent Kit v3.1 (10X Genomics). Sequencing was performed on an Illumina platform (NovaSeq), following the manufacturer’s instructions.

The Cell Ranger Analysis Pipeline (version 6.0.2) was used to create sequencing libraries from single-cell transcriptomes without specifying a targeted panel. After sequencing, the analysis pipeline takes the FASTQ files, a reference genome file (mouse: mm10), and a transcriptome annotation file (mouse: GENCODE vM23/Ensembl 98) for sequence alignment. The pipeline generates a unique molecular identifier (UMI) count matrix, which will be processed using the Python package Scanpy(1) (version 1.8) for further analysis. To remove low-quality cells, which is a major concern in microdroplet-based experiments, we filtered out cells with the UMI/gene numbers threshold (200 < gene numbers < 6000). Following a visual inspection of the distribution of cells by the fraction of mitochondrial genes expressed, we further discarded low-quality cells where >10% of the counts belonged to mitochondrial genes. After applying these QC criteria, 6380 single cells remained and were included in the downstream analyses. Library size normalization was performed with the pp.normalize_total function in Scanpy(1)to obtain the normalized counts. Specifically, the global-scaling normalization method normalized the gene expression measurements for each cell by the total expression, multiplied by a scaling factor (10,000 by default), pp.log1p function, and then log-transformed. Top variable genes across single cells were identified using the method described by Macosko et al (2). The most variable genes were selected using the pp.highly_variable_genes function in Scanpy (1). Principal component analysis (PCA) was performed to reduce the dimensionality of the dataset with the tl.pca function in Scanpy(1). Graph-based clustering was performed to cluster cells according to their gene expression profiles using the pp.neighbors function in Scanpy (1). Cells were visualized using 2-dimensional Uniform Manifold Approximation and Projection (UMAP) algorithms with the tl.umap function in Scanpy (1). We used the FindAllMarkers function (test.use = wilcox) in Seurat (3) to identify marker genes of each cluster. For a given cluster, FindAllMarkers identified positive markers compared with all other cells (Adjusted P value < 0.05 and |fold change| > 2 was set as the threshold of significant marker genes). Then, we used the R package g (4) to perform a functional enrichment analysis of marker genes. The marker genes for each cluster were mapped to known sources of functional pathways, and the hypergeometric distribution was used to check whether these biological processes were over-represented, including pathways from Gene Ontology (GO), KEGG, Reactome, WikiPathways, etc. The sequencing and bioinformatics analysis were performed by Genechem Co., Ltd. (Shanghai, China).

1. Wolf FA, Angerer P, Theis FJ. SCANPY: large-scale single-cell gene expression data analysis. Genome Biol 2018;19:15

2. Macosko EZ, Basu A, Satija R, Nemesh J, Shekhar K, Goldman M, et al. Highly Parallel Genome-wide Expression Profiling of Individual Cells Using Nanoliter Droplets. Cell 2015;161:1202-14

3. Butler A, Hoffman P, Smibert P, Papalexi E, Satija R. Integrating single-cell transcriptomic data across different conditions, technologies, and species. Nat Biotechnol 2018;36:411-20

4. Raudvere U, Kolberg L, Kuzmin I, Arak T, Adler P, Peterson H, Vilo J. g:Profiler: a web server for functional enrichment analysis and conversions of gene lists (2019 update). Nucleic Acids Res 2019;47:W191-W8
