# Supplementary material for: Pericytes recruited by CCL28 promote vascular normalization after anti-angiogenesis therapy through RA/RXRA/ANGPT1 pathway in lung adenocarcinoma
Source: J Exp Clin Cancer Res. 2024 Jul 29;43:210. doi: 10.1186/s13046-024-03135-3 (PMC11285179; doi:10.1186/s13046-024-03135-3)

Supplementary Fig. 1

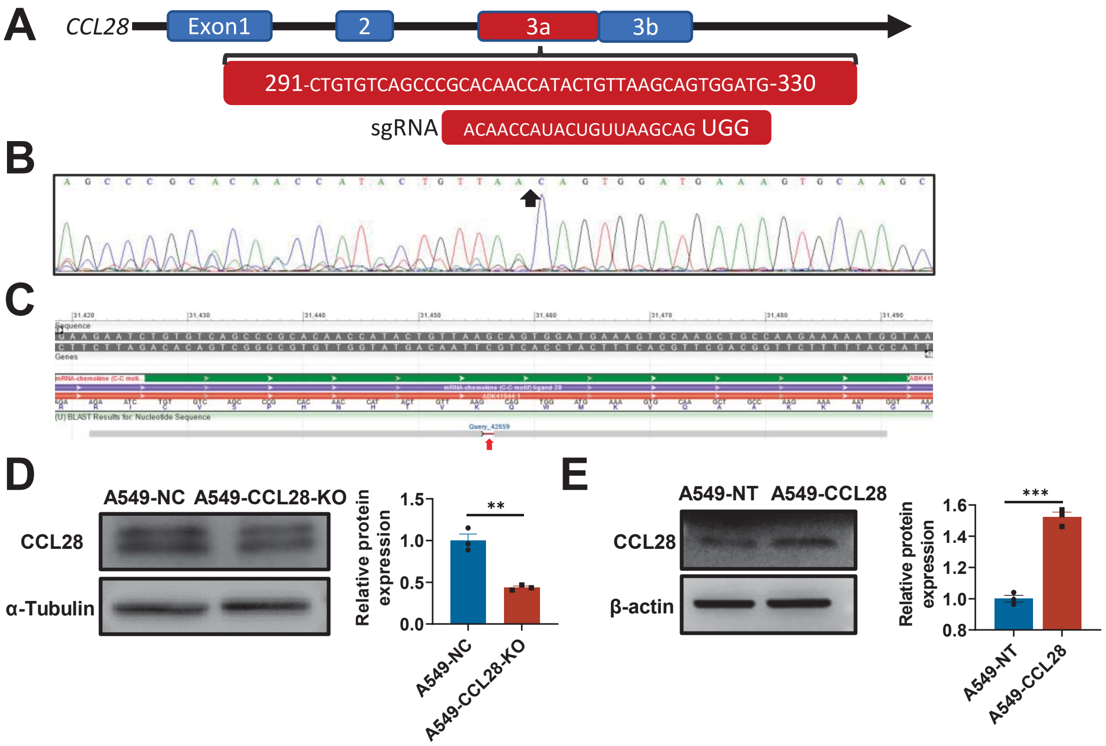

Supplementary Fig. 2

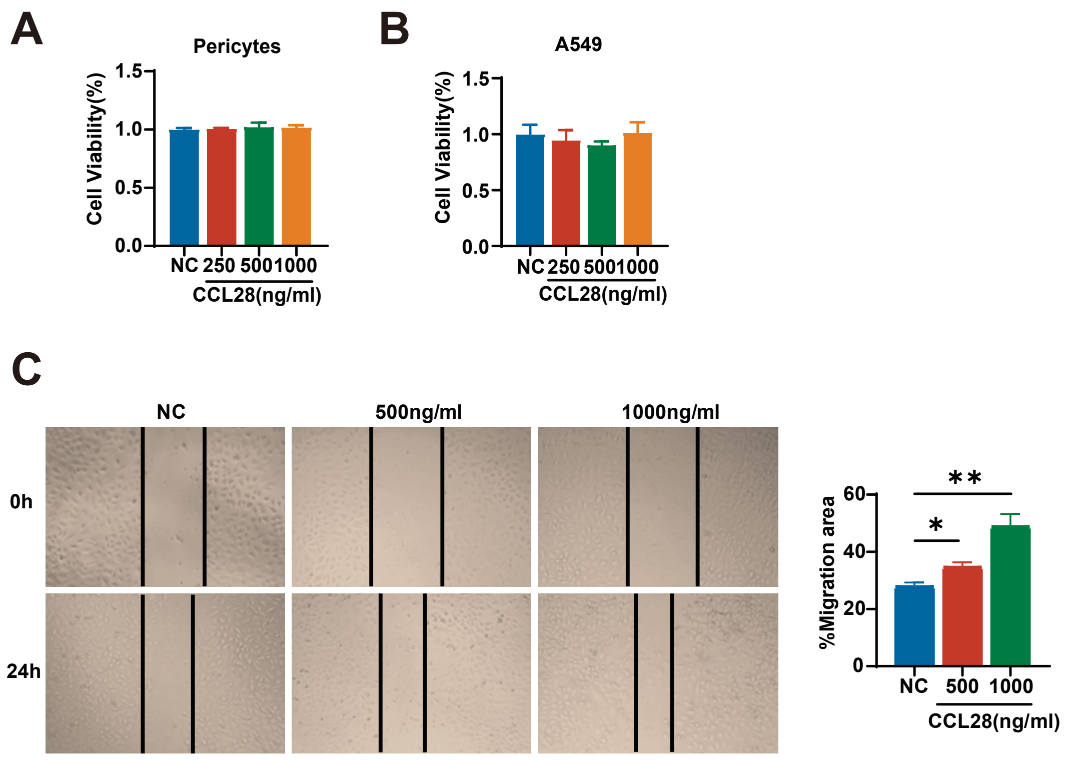

Supplementary Fig. 3

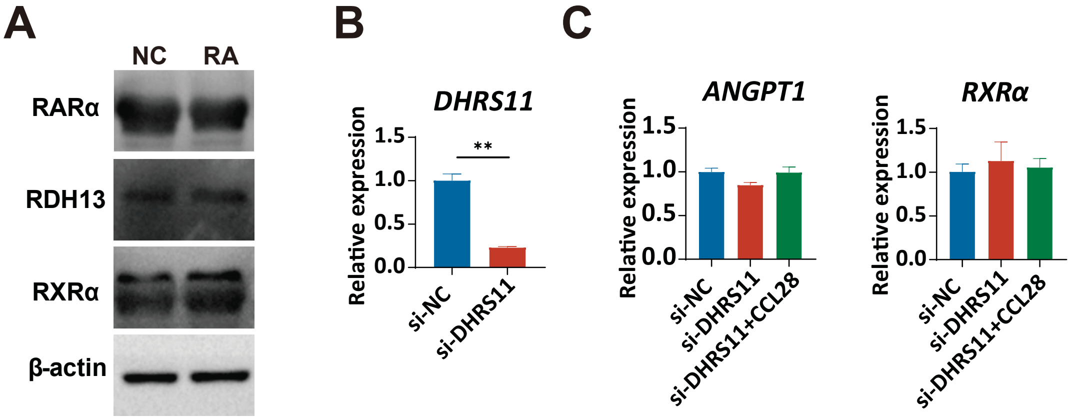

Supplementary Fig. 4

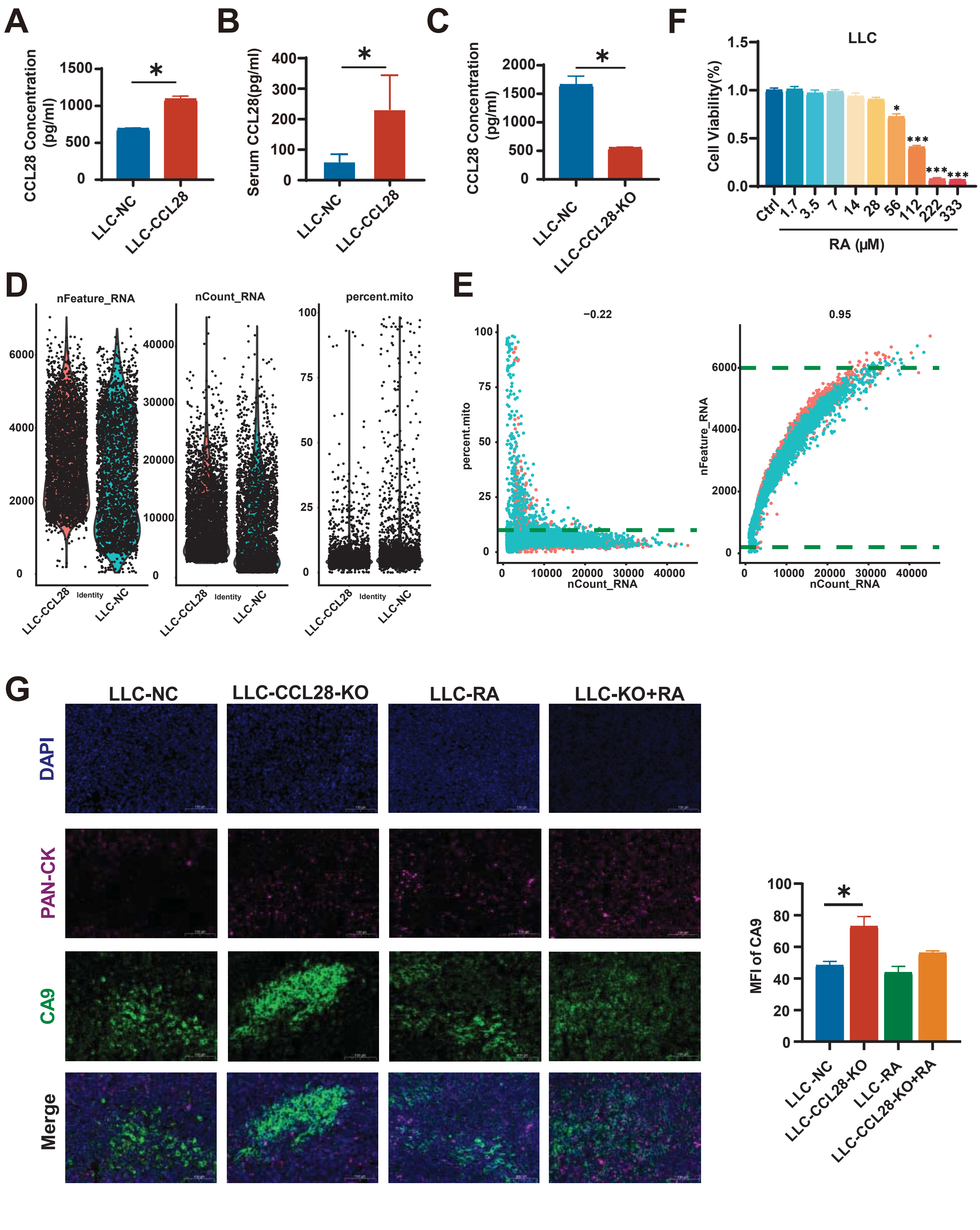

Supplementary Fig. 5

A

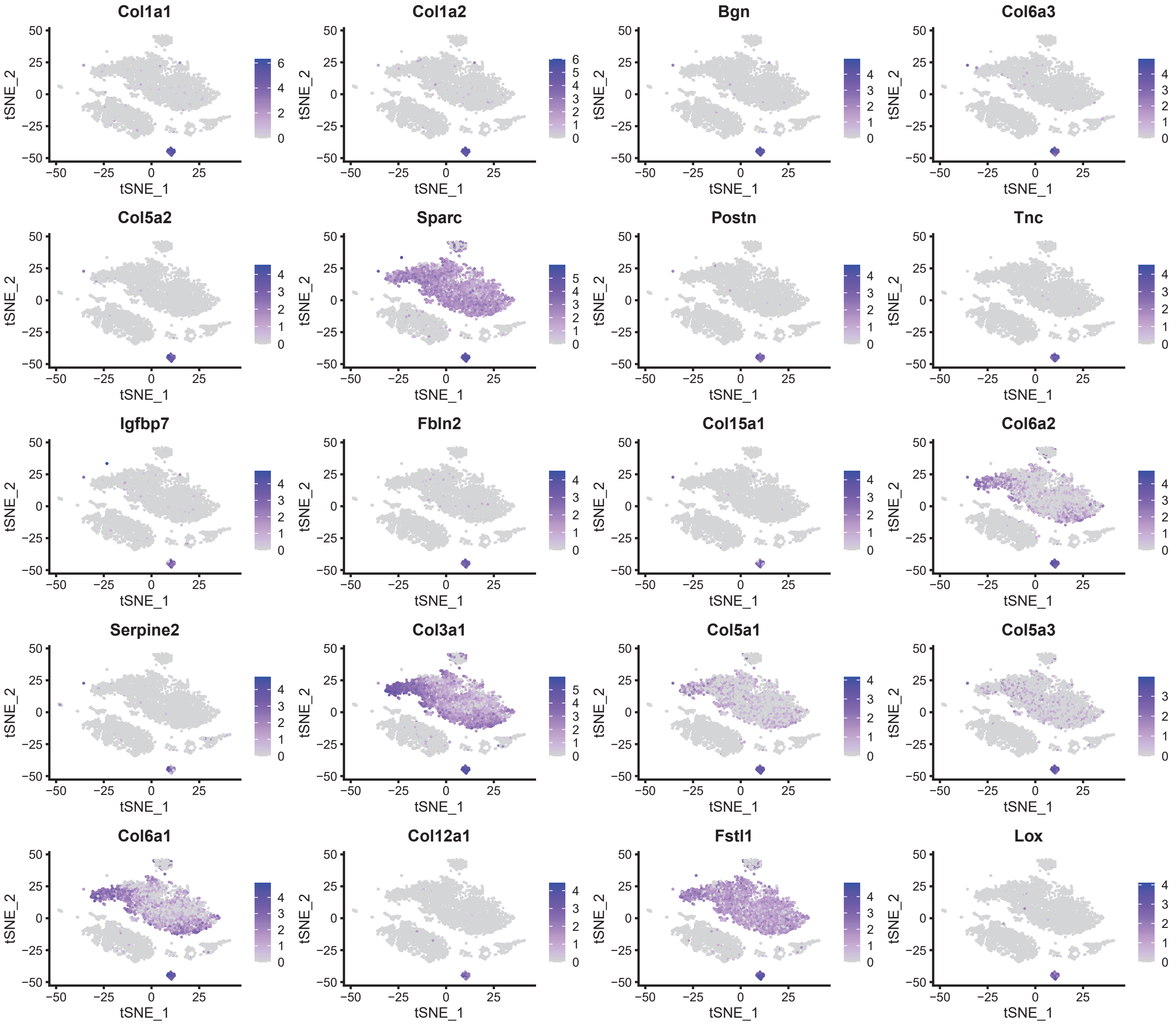

Supplement: Supplementary file 2 — Supplementary Material 2. [file 13046_2024_3135_MOESM2_ESM.pdf]
