# Supplementary material for: Pericytes recruited by CCL28 promote vascular normalization after anti-angiogenesis therapy through RA/RXRA/ANGPT1 pathway in lung adenocarcinoma
Source: J Exp Clin Cancer Res. 2024 Jul 29;43:210. doi: 10.1186/s13046-024-03135-3 (PMC11285179; doi:10.1186/s13046-024-03135-3)
Supplement: Supplementary file 3 — Supplementary Material 3. [file 13046_2024_3135_MOESM3_ESM.docx]

**Supplementary Table 1, prediction of transcription factors binding promotor of CCL28 gene**

| Hypoixa-responsive Transcription Factors* | Prediction Score** | Fold Change*** |
| --- | --- | --- |
| HIF-1/2A | NO | 0.91/1.14 |
| NFκB | NO | 1.07 |
| CREB | NO | 1.27 |
| AP-1 | NO | 1.50 |
| p53 | NO | 1.24 |
| **SP-1**/ SP-3 | **89.0**/NO | 0.92/0.98 |
| Egr-1 | NO | 0.70 |
| **CEBPB** | **93.7** | **1.52** |
| STAT5 | NO | 0.81 |
| *Cummins EP, Taylor CT. Eur J Physiol (2005) 450: 363–371;  ** Prediction with TFSEARCH ver.1.3;  *** Expression fold change under hypoxic condition. | | |
