# Supplementary material for: Pericytes recruited by CCL28 promote vascular normalization after anti-angiogenesis therapy through RA/RXRA/ANGPT1 pathway in lung adenocarcinoma
Source: J Exp Clin Cancer Res. 2024 Jul 29;43:210. doi: 10.1186/s13046-024-03135-3 (PMC11285179; doi:10.1186/s13046-024-03135-3)
Supplement: Supplementary file 4 — Supplementary Material 4. [file 13046_2024_3135_MOESM4_ESM.docx]

**Supplementary Table 2, correlation between expression of CEBPB and CCL28 in 44 lung adenocarcinoma cell lines**

| Sample ID in cBioPortal | CCL28 (mRNA expression (RNA Seq RPKM)) | CEBPB (mRNA expression (RNA Seq RPKM)) |
| --- | --- | --- |
| A549_LUNG | 0.10957 | 10.97104 |
| CALU3_LUNG | 1.08279 | 25.57125 |
| HCC1195_LUNG | 0.91848 | 12.70044 |
| HCC1833_LUNG | 0.825 | 8.03948 |
| HCC2279_LUNG | 0.87322 | 10.41143 |
| HCC366_LUNG | 1.63333 | 31.25001 |
| HCC4006_LUNG | 3.84063 | 9.96449 |
| HCC44_LUNG | 0.24629 | 7.13332 |
| HCC827_LUNG | 1.4722 | 6.55104 |
| HS229T_FIBROBLAST | 0.21953 | 14.88523 |
| HS618T_FIBROBLAST | 0.01906 | 11.13353 |
| NCIH1355_LUNG | 9.31634 | 30.8907 |
| NCIH1373_LUNG | 4.52789 | 8.79638 |
| NCIH1395_LUNG | 1.52578 | 9.23285 |
| NCIH1568_LUNG | 0.40405 | 11.28214 |
| NCIH1623_LUNG | 2.77049 | 23.16709 |
| NCIH1648_LUNG | 8.48295 | 35.08263 |
| NCIH1650_LUNG | 8.66735 | 35.11992 |
| NCIH1651_LUNG | 2.54022 | 12.9775 |
| NCIH1734_LUNG | 0.64216 | 16.73041 |
| NCIH1781_LUNG | 1.09535 | 22.67634 |
| NCIH1792_LUNG | 2.97586 | 10.37919 |
| NCIH1793_LUNG | 5.25301 | 42.69852 |
| NCIH1838_LUNG | 2.01375 | 16.8866 |
| NCIH1975_LUNG | 2.65172 | 10.06674 |
| NCIH2073_LUNG | 2.21661 | 9.79102 |
| NCIH2085_LUNG | 1.93643 | 12.5861 |
| NCIH2087_LUNG | 1.3142 | 9.39588 |
| NCIH2126_LUNG | 2.13122 | 29.24337 |
| NCIH2228_LUNG | 3.9956 | 36.0145 |
| NCIH2291_LUNG | 3.04416 | 13.53405 |
| NCIH2342_LUNG | 1.88292 | 10.11728 |
| NCIH2347_LUNG | 2.54865 | 8.3728 |
| NCIH322_LUNG | 0.59177 | 14.77056 |
| NCIH3255_LUNG | 1.67782 | 22.0441 |
| NCIH358_LUNG | 1.94408 | 11.70856 |
| NCIH522_LUNG | 0.77639 | 5.9561 |
| NCIH596_LUNG | 7.31307 | 59.35265 |
| NCIH647_LUNG | 1.31201 | 25.41829 |
| NCIH650_LUNG | 4.94142 | 30.81498 |
| NCIH838_LUNG | 3.11319 | 26.88646 |
| NCIH854_LUNG | 1.31638 | 10.85356 |
| RERFLCAD1_LUNG | 2.62973 | 16.34664 |
| RERFLCAD2_LUNG | 1.778 | 12.24629 |
